# Synthetic mutualism in engineered *E. coli* mutant strains as functional basis for microbial production consortia

**DOI:** 10.1002/elsc.202100158

**Published:** 2022-05-06

**Authors:** Tobias Müller, Simon Schick, Jonathan Beck, Georg Sprenger, Ralf Takors

**Affiliations:** ^1^ Institute of Biochemical Engineering University of Stuttgart Stuttgart Germany; ^2^ Institute of Microbiology University of Stuttgart Stuttgart Germany

**Keywords:** metabolic engineering, mutual auxotrophic dependency, spatially linked bioreactors, synthetic co‐culture, tryptophan

## Abstract

In nature, microorganisms often reside in symbiotic co‐existence providing nutrition, stability, and protection for each partner by applying “division of labor.” This principle may also be used for the overproduction of targeted compounds in bioprocesses. It requires the engineering of a synthetic co‐culture with distributed tasks for each partner. Thereby, the competition on precursors, redox cofactors, and energy—which occurs in a single host—is prevented. Current applications often focus on unidirectional interactions, that is, the product of partner A is used for the completion of biosynthesis by partner B. Here, we present a synthetically engineered *Escherichia coli* co‐culture of two engineered mutant strains marked by the essential interaction of the partners which is achieved by implemented auxotrophies. The tryptophan auxotrophic strain *E. coli* ANT‐3, only requiring small amounts of the aromatic amino acid, provides the auxotrophic anthranilate for the tryptophan producer *E. coli* TRP‐3. The latter produces a surplus of tryptophan which is used to showcase the suitability of the co‐culture to access related products in future applications. Co‐culture characterization revealed that the microbial consortium is remarkably functionally stable for a broad range of inoculation ratios. The range of robust and functional interaction may even be extended by proper glucose feeding which was shown in a two‐compartment bioreactor setting with filtrate exchange. This system even enables the use of the co‐culture in a parallel two‐level temperature setting which opens the door to access temperature sensitive products via heterologous production in *E. coli* in a continuous manner.

**Abbreviations**
ANT: anthranilate
CHOR: chorismate
TRP: tryptophan

## 1 INTRODUCTION

Over the past decade, we are witnessing an era of industrial transformation toward a rising importance of a multidisciplinary bioeconomy [1]. This reflects the need for establishing a circular economy utilizing sustainable substrates such as sugar from renewable sources [2, 3]. So far, such processes are dominated by axenic cultures. These cells are engineered to produce the targeted compounds in mono‐cultures. Often enough, this requires the equilibrated re‐arrangement of carbon fluxes and of reduction‐ and energy management via metabolic engineering strategies [4]. Although, a variety of diverse compounds can already be successfully synthesized using sophistically engineered production mono‐cultures, excessive adjustments of sensitive intracellular networks may result in a suboptimal microbial production system [5]. Often, there is a competition between precursors, redox cofactors and energy needed for growth versus the need for an optimal product formation.

A look at nature shows, that the commonly applied industrial workhorse microorganisms originate from ubiquitous consortia in their natural habitats. There, they act as part of basic interspecies interaction patterns within a complex biomatrix of diverse participants. The bilateral forms of interaction may be classified in asynchronous combinations of positive, negative, and neutral relations of parties (parasitism, commensalism, amensalism) or mutually beneficial or obstructive modes of action (mutualism or competition) [6]. For instance, syntrophic consortia show an essentially interdependent and mutually beneficial form of interaction. Through the interplay of a food‐chain‐like connection and thermodynamic advantages established for each partner, effective cooperation can be achieved for the mutual benefit [7]. The bridging of ubiquitous auxotrophies within cooperative microbial assemblies represents one of the central mechanisms of interaction in native microbial consortia [8]. Besides, mutual benefits are even known for the joint defense against enemies through provision of antibiotic compounds [9].

**PRACTICAL APPLICATION**

Consortia‐based microbial production strategies may be a key concept of future biotechnological applications. Optimally, new co‐culture platforms open up optimized synthesis strategies while ensuring straight forward handling, similar to current mono‐culture approaches. The presented synthetic co‐culture upholds stability throughout cultivation processes by its enforced mutualistic symbiosis. The bi‐lateral cross‐feeding based approach therefore comes close to the maxim of robust consortia operation. The co‐culture keeps its reciprocal dependency while the strains’ genomes could be further optimized individually to unlock advanced production strategies. Furthermore, the described filter‐coupled‐two‐compartment reactor system can be used to present specific environmental conditions to the cellular submodules. The provision of different temperature settings could beneficially influence co‐culture syntheses based on heterologous, for example, plant or fungi enzymes in microorganisms. Potentially, the temperature‐variable process strategy shown could be extended to other physio‐chemical parameters to enhance product formation in diverse future applications.

The rational transfer of knowledge gained from studies of native consortia to biotechnological co‐culture applications therefore provides a toolbox expansion for research and industry. In addition to the use of specialized (cross‐kingdom) consortia for the conversion of basic raw materials into biofuels such as ethanol or isobutanol in consolidated bioprocesses [10, 11, 12], the use of modularly engineered synthetic communities to produce value‐added bio compounds is shifting into the spotlight of interest [13].

For example, recent developments focused on the formation of derivatives of aromatic amino acids with recombinant *Escherichia coli* cultures. For instance, indigo, a pigment conventionally produced chemically for the textile industry, could be bio‐synthesized from simple carbon substrates applying an engineered *E. coli*–*E. coli* system [14]. Additionally, tryptamine synthesis was established in subsequent studies [15] and the biosynthesis of rosmarinic acid was greatly improved compared to mono‐culture performance [16] by using a consortium of three fine‐tuned *E. coli* submodules. By analogy, the production of the flavonoid flavan‐3‐ol was successfully achieved in a co‐culture of *E. coli* each harboring modules of the complex biosynthesis with well equilibrated redox cofactor needs [17].

All examples have in common, that the product of species A is used as substrate of species B where biosynthesis is completed. This unidirectional linkage between the species bears an intrinsic drawback: imbalanced substrate supply and not equilibrated growth rates may easily occur that deteriorate optimum product formation. However, industrial production conditions may be harsh imposing stress on the cells not only during bioproduction but also during the seed train. Hence, any new production strain, axenic or as co‐culture, should be robust enough to withstand the stress.

Consequently, this study focuses on the implementation of a tightly coupled cooperation between the partners of a co‐culture. Thereupon, a self‐controlling co‐culture should be created that allows easy handling similar to the well‐established axenic producer cells. The co‐culture should exploit the benefits of labor division [18, 19] based on metabolic cross‐feeding. Given that microbial hosts may be applied for the production of temperature sensitive (e.g., plant‐derived) recombinant or valuable therapeutic proteins [20, 21, 22], the approach should additionally consider the cultivation of the interacting partners in spatially separated compartments with different temperature levels.

To showcase our approach, we present a couple of engineered *E. coli* mutants capable of producing of aromatic amino acid derived products. Model guided design will be used to engineer the tryptophan (TRP) pathway split through gene deletions. Consequently, mutual interactions will be enforced leading to the division of labor in a tightly controlled co‐culture. Single‐ and two‐compartment tests will be shown illustrating the robustness of the consortium and its applicability in a spatially separated, two‐temperature level cultivation setting.

## 2 MATERIALS AND METHODS

### 2.1 Metabolic engineering and modeling

#### 2.1.1 Construction of auxotrophic strains derived from *E. coli* K‐12 wildtype strain LJ110

The strains and plasmids in this study are listed in Table 1. The primers are listed in the supporting information (Table S1). *E. coli* K‐12 wildtype (wt) strain LJ110 [23] was used as base for genetic modifications to create mutants for an auxotrophic co‐culture. The two main gene deletions (anthranilate [ANT] producer: Δ*trpD*; TRP producer: Δ*trpE*) were created by the plasmid‐based clustered regularly interspaced short palindromic repeats (CRISPR)/CRISPR‐associated (Cas) system of Jiang et al. [24]. Further strain modifications to improve the ANT/TRP production were gene deletions of *trpR* and *tnaA* that were performed with the recombineering method of Datsenko and Wanner [25]. Due to the nature of this method leaving an FRT‐flanked kanamycin cassette in the deleted gene locus, the TRP producer strain was transformed with the pCP20 plasmid [26] to remove its antibiotic resistance gene. The ANT producer kept its kanamycin resistance to be distinguished in later co‐culture experiments. All genetic changes (deletions) were verified by PCR. All strains were stored in glycerol stocks at ‐70°C.

**TABLE 1:** Strains and plasmids

**Strains and plasmids**	**Relevant genotype**	**Reference**
**Strains**		
*Escherichia coli*		
LJ110	W3110 *fnr^+^*	[23]
ANT‐1	LJ110 *ΔtrpD*	This study
ANT‐2	ANT‐1 *ΔtnaA::FRT*	This study
ANT‐3	ANT‐2 *ΔtrpR::FRT‐Km^R^‐FRT*	This study
TRP‐1	LJ110 *ΔtrpE*	This study
TRP‐2	TRP‐1 *ΔtrpR::FRT*	This study
TRP‐3	TRP‐2 *ΔtnaA::FRT*	This study
**Plasmids**		
CRISPR/Cas		
pCas	repA101(ts) km Pcas‐cas9 ParaB‐Red lacI^q^	[24]
pTarget‐trpD	*cat sgRNA‐ΔtrpD*	This study
pTarget‐trpE	*cat sgRNA‐ΔtrpE*	This study
Recombineering		
pKD46	*P_BAD_gam‐bet‐exo*	[25]
pCP20	*flp*	[26]
pCO1	*Km^R^*	[40]

#### 2.1.2 Stoichiometric network model and metabolic flux analysis

We utilized an *E. coli* network model which is composed of 155 metabolites and 157 reactions. The model stoichiometry was based on the *E. coli* reference model used by Schuhmacher et al. [27], which is essentially based on previously constructed models [28, 29]. Basically, the TRP biosynthetic pathway was refined for this work, and a TRP‐based production reaction was introduced. The model reconstruction as well as the flux balance analysis were performed within the Matlab (The MathWorks, Inc.) environment using the COBRA toolbox [30, 31]. Further processing details, model stoichiometry, flux balance analysis constrains, and calculated flux distributions can be found in the supplementary material.

### 2.2 Cultivation methods

All cultivations performed in the framework of this study were carried out in biological duplicates at least.

#### 2.2.1 Shaking flask cultivations

All strains in shaking flasks were cultivated in minimal medium (MM) [32] with 5 g L^–1^ glucose and 20 mg L^–1^ thiamin‐hydrochloride, adjusted with 1 mol L^–1^ HCl to a neutral pH value. Auxotrophic strains were further supplemented with 0.1 g L^–1^ TRP or ANT. Pre‐culture glass tubes with 5 mL MM were inoculated with a single colony of an agar plate of MM (+TRP or ANT, respectively) and incubated at 37°C and 200 rpm in an incubation shaker for 18 h (Infors AG, Switzerland). For the main culture (25 mL MM in a 250 mL shaking flask) cell suspension from the pre‐culture was washed with 500 μL MM to remove residual TRP or ANT before inoculating with an optical density measured at 600 nm (OD_600_) of 0.1. The incubation was performed at 37°C and 200 rpm for 24 h in an incubation shaker.

#### 2.2.2 Seed train and pre‐culture medium for bioreactor cultivations

The medium composition for pre‐culture growth was adapted from Albermann et al. [33]; it contained per liter medium: 3 g KH_2_PO_4_, 12 g K_2_HPO_4_, 5 g (NH_4_)_2_SO_4_, 0.1 g NaCl, 1.710 g sodium citrate ∙ dihydrate, 0.1125 g FeSO_4_ ∙ 7 H_2_O, 0.015 g CaCl_2_ ∙ 2 H_2_O, 0.3 g MgSO_4_ ∙ 7 H_2_O, and 0.0084 g thiamin ∙ HCl. A trace element solution [34] was added (0.4 mL L^–1^ for pre‐culture), which contained per liter: 2.8 g MnSO_4_ ∙ H_2_O, 2.5 g AlCl_3_ ∙ 6 H_2_O, 1.825 g CoCl_2_ ∙ 6 H_2_O, 0.5 g ZnSO_4_ ∙ 7 H_2_O, 0.5 g Na_2_MoO_4_ ∙ 2 H_2_O, 0.25 g CuCl_2_ ∙ 2 H_2_O, and 0.125 g H_3_BO_3_.

Glucose was added to reach 5 g L^–1^, TRP and ANT were added to compensate the respective genomic modifications (0.05 g L^–1^). The pre‐cultures of the kanamycin resistant strain ANT‐3 grew in the presence of 0.05 g L^–1^ kanamycin. 10% (w/w) HCl was used to set the pre‐culture medium pH to 7.2. The pre‐culture shaking flask cultures (50 mL in 500 mL shaking flask) were inoculated from a working cell bank (20% glycerol, stored at ‐70°C) of the respective strains and were cultivated on a shaking incubator at 37°C and 180 rpm. Before bioreactor inoculation, pre‐culture suspension was centrifuged (4°C, 7197 *g*, 5 min) and the pellet was resuspended in sterile 0.9% (w/v) NaCl. After another centrifugation step, the pre‐culture cell‐pellet was resuspended in the respective main culture medium.

#### 2.2.3 Single reactor co‐culture fermentations

The cultivations for the co‐culture dynamic analysis were done in a 1.5 L stainless steel stirred‐tank bioreactor in batch mode with a starting liquid volume of 0.7 L. The bioreactor was equipped with a single Rushton turbine and four baffles. 500 mL min^–1^ was set as the aeration rate. System overpressure was kept at 0.5 bar, and dissolved oxygen over 30% via a stirrer cascade control. Temperature was set to 37°C. The pH was kept at seven via controlled addition of 25% (w/w) ammonia solution and 25% (w/w) H_3_PO_4_.

The medium composition for the bioreactor cultivations was mainly the same as for the pre‐cultures, but with a 10 times higher trace element concentration. Also, no TRP or ANT was added. Furthermore, the medium was without antibiotics. The initial glucose concentration was set to 15 g L^–1^. Medium pH was set to 7 and 170 μL Antifoam J647 (Schill+Seilacher “Struktol” GmbH, Germany) per liter medium was added. Respective to the desired start optical density of OD_600_  =  0.1 and the targeted inoculation strain ratio of the co‐culture members, suspension was taken from the ANT‐3 and TRP‐3 pre‐cultures and processed as described above. Subsequently, the strains were combined to inoculate the reactor.

#### 2.2.4 Two compartment co‐culture cultivation

Two‐compartment fed‐batch fermentations were performed using two identically constructed stirred‐tank bioreactors as described above. To allow a transfer of filtered medium between the reactors, each reactor was equipped with an in situ filtration probe (D‐Series FISP, Rapid Flow 0.2 μm membrane; Flownamics, USA). They were connected with a peristaltic pump (120U; Watson‐Marlow Fluid Technology Group, England), enabling pressure driven filtrate discharge and transfer to the other bioreactor. The dead volume of the individual filtrate lines including filter modules were less than 3 mL. To check for possible cell transfer, samples were taken at selected process times for strain ratio analysis. The sampling point immediately before the start of filtrate exchange served as a mono‐culture control. Stirrer cascade control ensured dissolved oxygen levels above 30%. The aeration rate was set to 500 mL min^–1^, no overpressure was applied. The pH was kept at seven using 25% (w/w) ammonia solution. Starting temperature was set to be 37°C.

Reactor 1 was inoculated with the ANT‐3 pre‐culture to an OD_600_ of 0.1. The basal medium was the same as in single bioreactor tests. The total starting volume was 0.68 L, containing 4 g L^–1^ glucose and 0.0105 g L^–1^ TRP supplementation. Reactor 2 was inoculated with the TRP‐3 strain reaching a start OD_600_ value of 0.13 in 0.63 L. The initial glucose concentration was 8 g L^–1^ plus 0.021 g L^–1^ ANT. After 6.75 h of cultivation, filtrate exchange was started with a set exchange rate of 0.6 mL min^–1^. After 8 h, exponential glucose feeding (50 g L^–1^) started for reactor 2 to install the targeted TRP‐3 growth rate of 0.05 h^–1^ while temperature was set to 25°C. Later, after 11.9 h, exponential glucose feeding (100 g L^–1^) started in reactor 1 yielding ANT‐3 growth rate of 0.1 h^–1^ at 37°C.

### 2.3 Strain ratio identification

To resolve the co‐culture composition with respect to the individual strain proportions, samples were taken in sterile tubes, diluted with 0,9% (w/v) sterile NaCl and plated out on LB agar plates. After incubation at 37°C, 100 randomly picked single colonies were transferred to a LB + 0.05 g L^–1^ kanamycin agar plate. The fraction of cells able to form colonies on both plates represented the proportion of kanamycin resistant ANT‐3 in the consortium. Cells which only grew on the LB plate without kanamycin, were assigned to the TRP‐3 fraction. The procedure was done in duplicates for each sampling event.

### 2.4 Determination of the optical density, cell dry weight, and extracellular metabolite analysis

Optical density (OD_600_) was measured photometrically at 600 nm (DR3900, Hach‐Lange, USA) within the linear range of the photometer. Cell dry weight (CDW) values were derived from OD_600_ using the correlation (CDW [g L^–1^]  =  OD_600_ • 0.28; see supporting information). Sample supernatants were analyzed for quantification of extracellular ANT, TRP, and glucose concentration. TRP and ANT were quantified using UHPLC (UltiMate 3000‐Series; Dionex; Thermo Fisher Scientific Ind., USA) with a C‐18 column (Luna C18[2], 5 μm, 250×4.6 mm; Phenomenex Inc, USA) at 40°C. With a constant flow rate of 0.5 mL min^–1^, two mobile phases were used in a flow gradient protocol starting with 98% mobile Phase A (0,1% [v/v] Trifluoroacetic acid in HPLC grade water) and 2% mobile phase B (0,1% [v/v] Trifluoroacetic acid in pure methanol). During the first 2 min the ratio shifted to B: 20%, within 12 min to B: 60%, after 23 min to B: 77%, after 25 min to B: 98%, stayed at that ratio for 2 min and declined to B: 2% at 27.5–30 min. The supernatant samples were stored in a cooled autosampler (5°C) and a sample volume of 20 μl was injected. TRP was evaluated with UV detection at 280 nm, ANT related signals at 330 nm, quantification was based on external calibration (detectable linear range: 0.1–200 mg L^–1^). Glucose content was determined using commercially available enzyme kit (r‐biopharm AG, Germany).

## 3 RESULTS AND DISCUSSION

### 3.1 Design of a bi‐directional, mutualistic *E. coli*–*E. coli* co‐culture

The biosynthesis of aromatic amino acids either as products or as intermediates for follow‐up conversions is well studied in numerous reports [35]. In particular, compounds branching off from the TRP pathway are of commercial interest [36]. We chose TRP biosynthesis to showcase the feasibility of installing a robust co‐culture gaining stability by bi‐directional exchange of auxotrophic compounds. Noteworthy, auxotrophies need to be carefully engineered to fulfill a number of basic constraints. In essence, challenges are derived from the prospected application in commercial bioprocesses which is dominated by mono‐cultures so far. Industrial infrastructure for bioproduction is prepared for axenic fermentations, co‐cultures therefore are “the new kid on the block.” According to experience, this requires convincing new arguments to give co‐cultures a chance for commercial scale application.

1. As a prerequisite, the handling of co‐cultures should be as easy as the one for a mono‐culture. No additional care should be required to install proper ratios of the individual co‐culture species during production. Consequently, the ideal co‐culture system should possess an intrinsic mechanism of self‐control installing proper ratios automatically.
2. The implementation of individual optima for growth and production rates should be possible for each species in order to exploit the benefits of the co‐culture at best. Often, metabolic engineering toward a targeted product in a single host would have led to competing requirements of reduction equivalents, precursors, energy demands, etc. Hence, implementing separated tasks in multiple species of a co‐culture may prevent such conflicts leading to the improved production of the target compound in the co‐culture. Apparently, the co‐culture should be designed such that the individual optima for growth, substrate uptake, and product formation should be implementable in bioprocesses.

Considering the constraints (1) and (2) it was the goal of this study to create a fundamental co‐culture structure showcasing the exploitation of TRP. The latter might serve as a proxy for related products. The realization of the concept requires the identification and engineering of ideal cell‐to‐cell interactions which was done by the creation of mutual dependencies. With regard to constraint (2) the synthetic co‐cultures should be exposed to different bioreactor compartments with individual growth and production conditions. In the second part of this study, we exemplify the scenario of installing a maximum growth, high temperature (37°C) condition in compartment 1 which is connected with a low growth, low temperature (25°C) compartment 2. The setting may serve as an exemplification for the production of fungi or plant derived products in compartment 2 which—often enough—require low temperatures to meet the related enzyme activity optima. It is assumed that related heterologous genes are expressed in *E. coli*.

#### 3.1.1 Model guided strain engineering of the co‐culture

**FIGURE 1:**
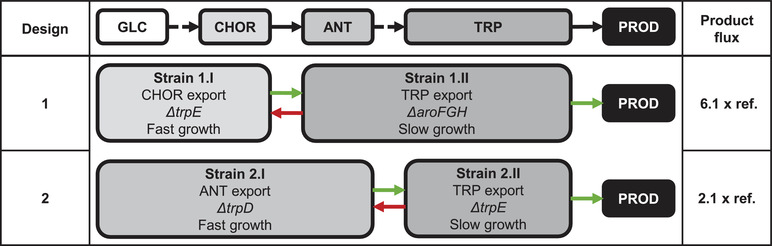
Simplified comparative representation of the flux balance analysis models of different tryptophan‐based product‐synthesizing co‐cultures, which differ in design by the different deletions introduced and metabolites cross‐feed. In the top line, a coarse linear pathway to the target product is drawn. This overall pathway is taken up in the co‐culture designs shown, in that way that the two co‐culture partners cover the respective area of the simplified pathway. Information about key knockouts, growth characteristics, and cross‐feeding related properties are indicated. Furthermore, green arrows represent the maximization constraints of the linear programming of the FBA. The red arrows indicate set constraints. Additional fixed boundary conditions were set with respect to growth rates and glucose uptake rates. A forced energy sink was incorporated as a cell maintenance representation. Exact values of the set constraints can be taken from the supplementary information (Table S3). The comparative data refer to the production flux relative to a reference mono‐culture without deletion

Using the stoichiometric model of *E. coli* (see supporting information) flux balance analysis were performed to compare the performance of a co‐culture producing a hypothetical TRP‐derived compound with the biosynthesis in a mono‐culture. Notably, simulations considered pre‐defined rates for growth and substrate uptake which were deemed best for the envisioned scenario of low temperature TRP formation by the co‐culture (see appendix). In addition, the cross‐feeding related TRP allocation was taken into account. For comparison, the mono‐culture performance was simulated considering equal production conditions Two hypothetical co‐culture compositions were considered that differ by deletions and pathway splits, which are presented in Figure 1.

Design 1 (see Figure 1) assumed a split at chorismate (CHOR) to create a mutual dependence. Strain 1.II completely lacks the shikimate pathway by *ΔaroFGH* which creates a theoretical CHOR auxotrophy. Strain 1.I is characterized by *ΔtrpE* preventing CHOR conversion to ANT. Consequently, strain 1.I is TRP auxotrophic. For evaluating the strain design, CHOR and TRP production was maximized for strains 1.I and 1.II, respectively. As indicated in Figure 1, TRP derived production rate via the CHOR‐auxotrophic strain was 6.1‐fold higher in the co‐culture than in the mono‐culture.

**FIGURE 2:**
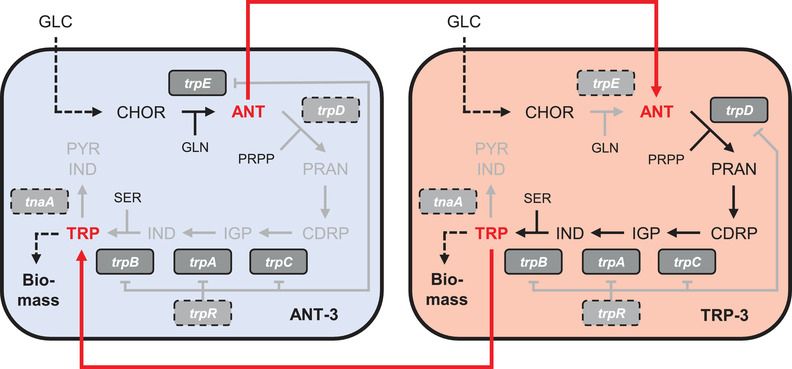
Schematic principle of the auxotrophic co‐culture and crossfeeding between the strains ANT‐3 and TRP‐3 (gray color/dashed frame = depleted pathways or genes). GLC, glucose; CHOR, chorismate; GLN, glutamine; ANT, anthranilate; PRPP, phosphoribosyl pyrophosphate; PRAN, phosphoribosyl anthranilate; CDRP, 1‐(o‐carboxyphenylamino)‐1‐deoxy‐ribulose 5‐phosphate; IGP, indoleglycerol 3‐phosphate; IND, indole; TRP, L‐tryptophan; SER, L‐serine; PYR, pyruvate

The benefit of the optimal division of labor becomes clear when looking at the co‐culture flux distributions. The fast growing strain 1.I exported over 10‐time more CHOR than the slow growing mono‐culture would be able to synthesize itself. Thus, significantly more CHOR was available for the TRP producer strain 1.II in the co‐culture setup. However, the TRP synthesizing strain could only metabolize 71% of the precursor provided by the partner strain. The reason was, that the further conversion of CHOR is also linked to the availability of additional reactants of the metabolic pathway. As shown in Figure 2, glutamine, PRPP, and serine is required to convert the supplied CHOR into TRP. Through deletion enforced flux redistribution, precursors, and co‐factor availabilities are significantly increased in strain 1.II compared to the mono‐culture reference. The direct synthesis fluxes of glutamine, serine, and PRPP were increased by a factor of five in strain 1.II compared to the mono‐culture. NADPH and ATP provision exceeded the mono‐culture 1.4 and 1.6 times, respectively (an excerpt of the flux distributions can be found in the supplementary information, Table S4). Thus, while in the mono‐culture case the flux into the CHOR pathway per se was the limiting factor for TRP synthesis, the co‐culture performance was restricted by the conversion of CHOR into TRP, which, however, was at significantly increased levels.

Unfortunately, this strategy remains theoretical as *E. coli* does not support CHOR import [37]. Therefore, the focus for further strain engineering was set on another biologically feasible co‐culture architecture. Design 2 follows the similar mindset of design 1 but considers strain 2.I as an ANT‐ and strain 2.II as a TRP producer. (Figure 1, Design 2). Again, strain 2.I was designed as TRP auxotrophic by deleting *trpE* whereas ANT auxotrophy was implemented in strain 2.II by partly removing *trpD*. The first prevents synthesis of ANT from CHOR, the second removes the ability to convert ANT into phosphoribosyl anthranilate (PRAN, see Figure 2). As indicated in Figure 1, this co‐culture design generated more than double of the expected production flux compared to using a single host. The comparison of flux patterns reveals the same rationale as for the CHOR case basically identifying increased precursor and co‐factor availability as the core reason for superior performance of the co‐culture (See Table S4). Consequently, the dry‐lab construction of co‐culture design 2 was realized by strain engineering and further tested in wet‐lab approaches.

#### 3.1.2 Advanced genetic engineering approaches addressing TRP control and degradation

The first generation of interacting *E. coli* strains, numbered as “‐1,” was tested on several media. Theoretically, both auxotrophic strains should not be able to grow on minimal medium (MM) without a TRP or ANT source. This could be verified on MM agar plates as both strains did not form colonies if streaked out separately. However, when both strains were streaked out on the same MM agar plate (without crossing), a zone of bacterial growth was detected where the two cultures came close (see supporting information Figure S1). Apparently, ANT‐1 produced and secreted a compound (most likely ANT) into the surrounding medium. This led to growth of TRP‐1 which in turn converted the compound into TRP. The latter fed ANT‐1 leading to detectable growth of both microorganisms.

We next wanted to improve ANT and TRP exchange and therefore removed reactions which either compete for TRP (tryptophanase) or limit TRP formation on the transcriptional level (Trp repressor). Thus, the genes *tnaA* (for tryptophanase) and *trpR* (encoding the Trp repressor) were successively deleted (see Figure 2). This resulted in strains ANT‐3 and TRP‐3 that were further used in the study.

### 3.2 Co‐culture achieving axenic wildtype‐like growth in shaking flasks

**FIGURE 3:**
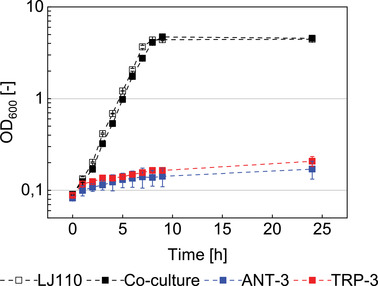
Growth behavior of the ANT‐3 – TRP‐3 co‐culture compared to the LJ110 wildtype, OD_600_ (in log10) plotted against the time. Controls: ANT‐3 and TRP‐3 in single culture without additional supplementation. Error bars indicate standard deviation of biological replicates

Metabolic engineering sometimes causes growth impairment, especially when dealing with essential genes. Comparing the growth behavior between the auxotrophic co‐culture and the original *E. coli* LJ110 wildtype offered deeper insight. Figure 3 shows the results of the growth experiment in a minimal medium.

As depicted in Figure 3, the *E. coli* LJ110 wildtype (as control) grew exponentially. In contrast, the single‐cultured strains ANT‐3 and TRP‐3 showed no growth after about one doubling of the OD_600_. Though they reached OD_600_ of ∼ 0.2 after 24 h, this may be explained by stored intracellular TRP from the pre‐culture, which was not completely removed by the washing step. Together with the phenotype of MM agar plates, this result in liquid medium is qualified as a proof for the existing auxotrophies. As additional controls, the auxotrophic strains were supplemented with ANT or TRP in MM shaking cultures, respectively, showing wildtype like growth (data not shown).

When ANT‐3 and TRP‐3 were co‐cultured, they achieved a wildtype‐like growth progression. The synthetic co‐culture reached a maximum growth rate of μ_Co‐Culture_ = 0.56 ± 0.01 h^–1^ which is quasi equal to the growth of the wildtype strain (μ_LJ110_ = 0.57 ± 0.00 h^–1^) between 2 and 7 h. In conclusion, cross‐feeding should be sufficiently strong preventing any shortness of the auxotrophic compounds. Theoretically, TRP auxotrophy of ANT‐3 may be compensated by the uptake of phosphorylated intermediates of the TRP pathway, indole derivatives, or indole itself. However, the secretion of such phosphorylated compounds by TRP‐3 was deemed unlikely. Simple diffusive exchange of indole is possible [38], but no indole was detected by Kovac test (data not shown). Furthermore, since the tryptophanase coding gene *tnaA* has been deleted in both strains, avoiding TRP degradation to indole. Consequently, ANT and TRP exchange is still considered as the key metabolite exchange in the co‐culture.

### 3.3 Analysis of the co‐culture dynamics in joint cultivation reveals promising consortia traits

**FIGURE 4:**
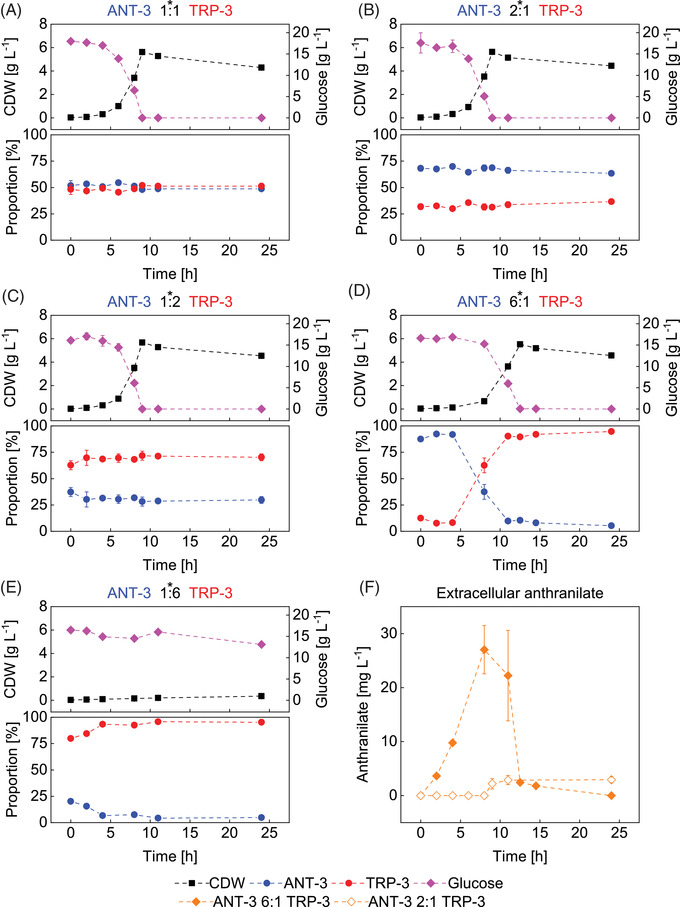
Assessment of the growth‐ and metabolite exchange behavior of the ANT‐3 – TRP‐3 co‐culture system when cultivated together in a batch approach at different relative initial ratios of the partner strains. The subfigures A‐E show both the biomass‐ and glucose concentration over time, as well as the relative proportion of the two strains ANT‐3 and TRP‐3 over the course of cultivation. The data marked with an asterisk refer to the planned experimental setting of the strain ratio at inoculation. Subgraph F shows the measured extracellular anthranilate concentrations of the experimental ANT‐3 – TRP‐3 6:1 and 2:1 approaches. No measurable anthranilate could be detected for the other strain ratios; therefore, the data are not shown. Also, no tryptophan was detectable for all given experimental setups. Error bars indicate standard deviation of biological replicates

Further insights in the interaction characteristics of the two strains were gained by cultivating *E. coli* ANT‐3 and TRP‐3 together in a single bioreactor. The aim was to investigate the effects of different inoculation ratios of ANT‐3:TRP‐3 on the performance of the co‐culture. Figure 4 depicts the deconvolution of subpopulation dynamics based on measurements of the total culture followed by plating tests as described in Section 2.

If balanced or moderately imbalanced ratios of the two partner strains were initially installed (ANT‐3:TRP‐3 of 1:1, 1:2, and 2:1) the co‐cultures showed very similar growth rates compared to the wildtype *E. coli* LJ110 (μ_LJ110_ = 0.63 ± 0.00 h^–1^; See Figure S2A). Growth rates of μ_1:1_ = 0.63 ± 0.00 h^–1^, μ_2:1_ = 0.61 ±0.02 h^–1^, and μ_1:2_ = 0.62 ±0.03 h^– 1^ were observed as depicted in Figure 4A–C. Interestingly, the initial strain ratios were maintained which reflects equal growth rates of each partner under these conditions. The finding represents a quality feature of good robustness. In essence, it labels the range of flexible inoculation ratios still achieving similar co‐culture performance.

A different picture was seen when the inoculation ratios of the partner strains were more imbalanced. The starting condition of a strongly increased fraction of ANT‐3:TRP‐3 of 6:1 led to a significantly reduced growth of the total population of μ_Co‐ culture_ = 0.45 ± 0.00 h^–1^. In contrast to the stable subpopulation conditions of Figure 4A–C, strong dynamics were found for ANT‐3:TRP‐3 of 6:1 (Figure 4D). During the first 4 h, the strain ratios remained stable while the extracellular ANT concentration increased (Figure 4F). Noteworthy, similar increases in ANT concentrations did not occur in any of the other co‐cultures (data not shown). After 4 h, the strain ratio flipped, finally installing the inverse ratio with dominating TRP‐3 and a minority of ANT‐3. This coincided with the complete consumption of ANT.

The subpopulation and ANT dynamics may be explained as follows: In the beginning, the dominating ANT‐3 population provided a surplus of ANT which was not consumed by the TRP‐3 subpopulation as fast as it was produced by the ANT‐3 species. As a consequence, ANT concentrations rose while the TRP‐3 strains could consistently access the metabolite necessary for growth. However, the TRP supply of the small TRP‐3 subpopulation for the relatively large number of ANT‐3 cells was not sufficient to provide enough TRP for this subpopulation. The resulting growth retardation created a growth advantage for the TRP‐3 cells that overgrew the ANT‐3 subpopulation until the declining ANT supply also limited growth of TRP‐3 cells. Whether or not the resulting ANT‐3:TRP‐3 strain ratio would remain stable could not be completely elucidated within this series of batch studies. Glucose supply became limiting in the cultivations which hampered further growth per se. However, the findings shown in Figure 4E anticipate that a stable but non‐functional subpopulation constellation has been reached.

Installing an ANT‐3:TRP‐3 ratio of 1:6 (Figure 4E), it was found that the total culture is barely able to grow (Figure 4E). During the first 4 h, the relatively small ANT‐3 subpopulation seemed to be able to provide enough ANT enabling TRP‐3 to grow slowly. However, the latter apparently produced only TRP for its own biosynthesis and did not secrete a surplus of TRP into the medium. Accordingly, ANT‐3 showed almost no growth, and the ratio shifted more to the side of TRP‐3. This deteriorated the growth conditions for both strains further, finally installing a non‐functional co‐culture similar to the result of Figure 4D. As a complementing observation, no extracellular ANT or TRP could be detected during the entire cultivation period.

The analysis of different inoculation ratios of ANT‐3 versus TRP‐3 indicates that stable subpopulation conditions can be easily installed provided that the ratios of the partners only vary within the range from 2:1 until 1:2. If the constraint is fulfilled, optimal mutual supply of nutrients is ensured enabling wildtype like growth kinetics for both strains. Virtually no accumulation of extracellular metabolites was observed which demonstrates the well equilibrated production and uptake of the auxotrophic compounds. The finding is remarkable as it means that such co‐cultures should be applicable for commercial application. The given window of operation ranging from 2:1 to 1:2 actually represents an easy‐to‐realize condition in industrial seed trains which are even more trimmed to deliver the same quality of mono‐cultures for each fermentation.

The investigation of the improper ANT‐3:TRP‐3 inoculation ratios of 6:1 or 1:6 revealed another remarkable result: Both scenarios finally ran into the same non‐functional co‐culture with a dominating TRP‐3 subpopulation. Interestingly enough, it was found that ANT‐3 can produce ANT even without being able to grow (Figure 4D). This opens the door for the future investigation of novel applications considering non‐growing ANT‐3 cells feeding a growing TRP‐3 subpopulation. Apparently, the latter is not able to produce TRP without ANT supply and thus cannot initiate the feeding and ultimately growth cycle.

### 3.4 Successful implementation of a two‐compartment reactor setup, cross‐linked via filter systems for spatially separated co‐culture application

As the fundamental suitability and functionality of the synthetic co‐culture was successfully shown, a further step of co‐culture testing was made: the application of the interacting strains in two intertwined compartments each running under different operating conditions.

As outlined above, the basic setting of the synthetic co‐culture reflects an anticipated application of the TRP producing strain for the formation of plant or fungi derived products. The latter are expected to be accessed via the heterologous amplification of related genes using TRP as a precursor for bioproduction. Because fungi and plants enzymes are likely to show enzyme optima at relatively low temperatures (compared to *E. coli* which are mesophilic), the showcasing production of TRP should happen at 25°C in compartment 2. Noteworthy, compartment 1, harboring the ANT producer should run under optimum growth conditions, that is, 37°C.

**FIGURE 5:**
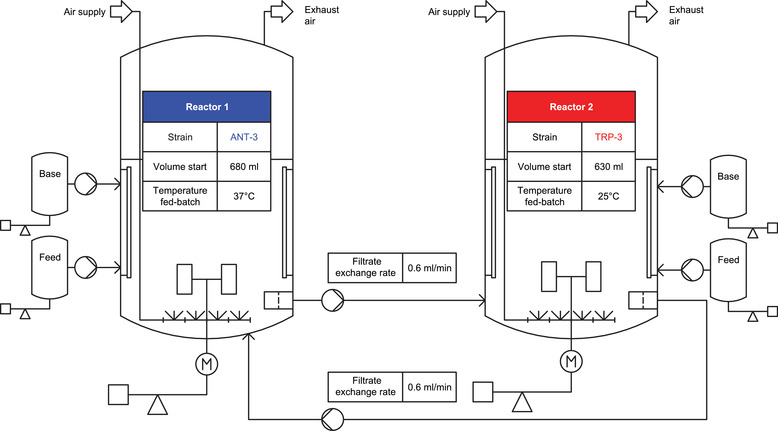
Technical representation of the double reactor system. Shown is the simplified setup of the two‐compartment reactor system used. The two reactors each harbored the indicated strain. Both reactors had in situ filter modules mounted in a side port. Peristaltic pumps were used to enable filtrate exchange between the reactors. Details regarding process conditions and process operation can be found in Section 2.2.4

It was necessary to ensure metabolite exchange between the reactors to comply with the auxotrophic dependencies of the two strains. This was achieved by implementing a filter system with minimal dead volume in both reactors. For this purpose, cylindrical ceramic filter modules were inserted into the reactors via side ports. The membranes were flushed tangentially by the agitated liquid flow, while a radially acting pressure difference was installed by the connected peristaltic pumps. Said filtration units enabled in situ filtrate removal retaining the cells in each compartment. The experimental setup is given in Figure 5.

**FIGURE 6:**
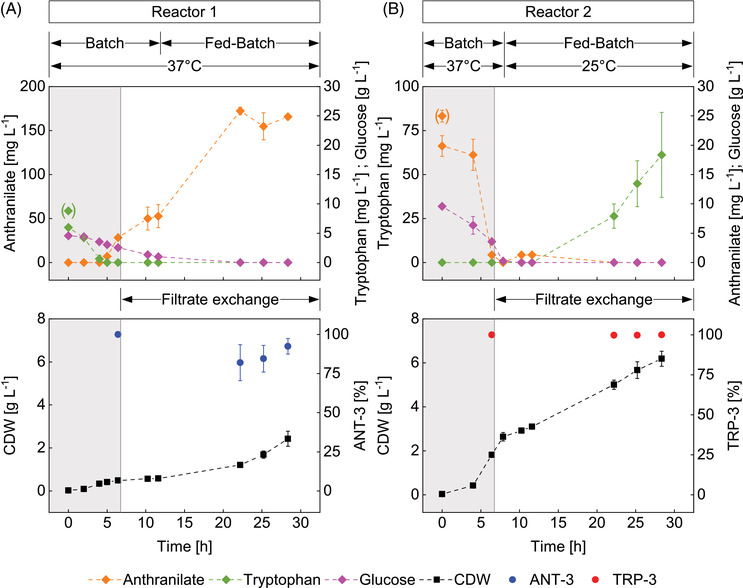
Representation of biomass and metabolite profiles of a co‐culture of auxotrophic strains, separated on two reactors, cross‐fed via a filter system‐mediated filtrate exchange between the reactors. (A) The strain ANT‐3 introduced in reactor 1 is tryptophan auxotrophic. (B) Strain TRP‐3 introduced in reactor 2 is anthranilate auxotrophic. The gray colored areas define the time before the start of the filtrate exchange between the reactors. The time periods of process‐relevant phases are marked for both reactors. The concentration curves of the extracellularly determined key metabolites anthranilate and tryptophan are shown, as well as the glucose course and the course of biomass development in the reactors together with the composition of the cultures. The values in brackets correspond to the respective concentrations in the medium before inoculation. Error bars indicate standard deviation of biological replicates

During the first phase of cultivation (regions shaded in gray, Figure 6), both strains were entirely dependent on the auxotrophic metabolites provided initially, as filtrate exchange started not earlier than 6.75 h of process time. It should also be noted that an immediate, cell‐associated decrease of the concentrations of TRP and ANT was observed after inoculation (bracketed value corresponds to the value before inoculation). While TRP‐3 in reactor 2 did not provide extracellular TRP during this initial phase, ANT‐3 in reactor 1 already produced excess ANT after TRP was depleted. The phenotype was already observed in the preliminary experiments (see previous section) and may be explained as follows: Because TRP is depleted, the allosteric TRP‐mediated feedback inhibition of ANT synthase is absent which allows enhanced production of ANT.

Apparently, TRP supply for ANT‐3 remained limiting. ANT‐3 cells continued showing rather low growth rates but enhanced ANT production during the early period of filtrate‐exchange based cultivation. The ANT transferred from reactor 1 to reactor 2 was almost completely consumed by the ANT auxotrophic TRP‐3. The extracellular ANT concentration in reactor 2 showed only a small intermediary accumulation. Furthermore, no residual glucose was found, indicating that the desired carbohydrate‐limited conditions were achieved with simultaneous production of TRP in bioreactor 2. In return, the transfer of the produced TRP to bioreactor 1 successfully closed the cross‐feeding loop between the two reactor compartments.

The maximal volumetric and biomass‐specific TRP production rates were Q_TRP,Max_ = 9.5 ± 3.6 mg_TRP_ (L_Reactor 2_ h)^–1^ and q_TRP,Max_ = 1.7 ± 0.7 mg_TRP_ (g_TRP‐3_ h)^–1^, respectively. The integral TRP/glucose yield over the process phase with filtrate exchange was Y_TRP/Glucose_ = 2.5 ± 1 mg_TRP_ g_Glucose,overall_^–1^. The results obtained were taken as evidence that the proof‐of‐concept works. In subsequent steps, TRP‐derived target compounds requiring low production temperatures can be addressed.

While the filtrate transfer from reactor 1 to reactor 2 was effectively cell‐free, the examination of the strain ratios gave evidence for the co‐existence of ∼15% TRP‐3 in bioreactor 1. This occurred about 10 h after filtration start (Figure 6) and is supposed to mirror insufficient cell retention via the filtration probe. The small subpopulation of TRP‐3 likely supported the TRP dependent growth of ANT‐3. Interestingly enough, the population shift as in Figure 4D did not occur. Because of glucose limited feeding, growth rates of the two strains converged diminishing potential individual growth advantages. Furthermore, TRP was additionally provided via the permeate of bioreactor 2 which supported the growth of ANT‐3.

Accordingly, the proof‐of‐concept of the two‐compartment setting applying the synthetic co‐culture was achieved. Future studies may either focus on the implementation of alternate filtration units, or rather contrarily, may install a co‐culture in bioreactor 1 on purpose for optimum ANT supply for bioreactor 2. This approach may ask for a fine tuning of inoculation ratios to consider the impact of different temperature levels on growth rates that was not considered yet in the co‐culture tests of Figure 4.

## 4 CONCLUDING REMARKS

Microbial consortia offer a high potential to distribute labor in synthetically designed co‐cultures, even enabling highly complex biosynthesis by exploiting individual advantages. In this study, two *E. coli* K‐12 strains’ truncated TRP pathways were metabolically engineered creating a mutually auxotrophic and cross‐feeding dependent co‐culture (ANT‐3 and TRP‐3). The model guided design of the co‐culture leads to a microbial consortium that showed high robustness in batch tests with well equilibrated exchange of auxotrophic compounds. Only extreme starting ratios of ANT‐3 versus TRP‐3 (1:6 or 6:1) resulted in non‐functioning co‐cultures with a dominant TRP‐3 strain.

The finding is remarkable as it reflects the robustness of the co‐culture with respect to variations in the seed train. It underlines the potential of applying this concept in prospective industrial applications that often follow the “KISS” principle—keep it simple and safe. Besides, the observations in the two‐compartment bioreactor setting outline that stable strain ratios could even be achieved in dominating ANT‐3 versus TRP‐3 cultures if proper glucose limitation is installed. Thereupon, multiple possibilities of alternate operational modes are conceivable comprising the concomitant use of a co‐culture and a mono‐culture in two interconnected compartments running under different conditions. Interestingly, ANT‐3 revealed high productivity in resting mode which expands the range of future application even further. Thus, co‐culture strategies with resting ANT producers and growing ANT converting cells could be a conceivable approach. Regarding TRP‐producing strains, next engineering approaches could offset the still existing TRP regulatory mechanisms (e.g., via *trpL* and allosteric feedback inhibition) and improve the TRP export [39].

The structural compartmentalization of this co‐culture opens the door to temperature‐sensitive bioprocesses, targeting the production of plant or fungi proteins that often possess low temperature optima. Not only batch but also continuous bioproduction should be possible thereby increasing the intrinsically low productivities in low temperature bioprocesses.

## NOMENCLATURE

**Table:** 

Q	[mg (L h)^–1^]	Volumetric production rate
q	[mg (g h)^–1^]	Biomass specific production rate
Y	[mg g^–1^]	Yield coefficient; product generated per glucose consumed
**Greek symbols**		
μ	[h^–1^]	Specific growth rate
**Indices**		
Max	Maximal rate	
n:m	Strain ratio of ANT‐3 (n) to TRP‐3 (m) in co‐culture	
600	Wavelength at 600 nm	

## CONFLICTS OF INTEREST

The authors have declared no conflicts of interest.

## Supporting information



SUPPORTING INFORMATION

## Data Availability

The data that support the findings of this study are available from the corresponding author upon reasonable request.
