# Supplementary material for: Synthetic mutualism in engineered E. coli mutant strains as functional basis for microbial production consortia
Source: Eng Life Sci. 2022 May 6;23(1):e2100158. doi: 10.1002/elsc.202100158 (PMC9815082; doi:10.1002/elsc.202100158)
Supplement: Supplementary file 1 — SUPPORTING INFORMATION [file ELSC-23-e2100158-s001.pdf]

Supporting information

Synthetic mutualism in engineered *E. coli* mutant strains as functional basis for microbial production consortia

Tobias Müller[1*]

Simon Schick[2*]

Jonathan Beck[1]

Georg Sprenger[2]

Ralf Takors[1]

[1] Institute of Biochemical Engineering, University of Stuttgart, Stuttgart, Germany

[2] Institute of Microbiology, University of Stuttgart, Stuttgart, Germany

*Tobias Müller and Simon Schick contributed equally.

Correspondence: Prof. Ralf Takors (ralf.takors@ibvt.uni-stuttgart.de). Institute of Biochemical Engineering. University of Stuttgart, Allmandring 31, 70569 Stuttgart, Germany.

Construction and analysis of auxotrophic *E. coli* strains

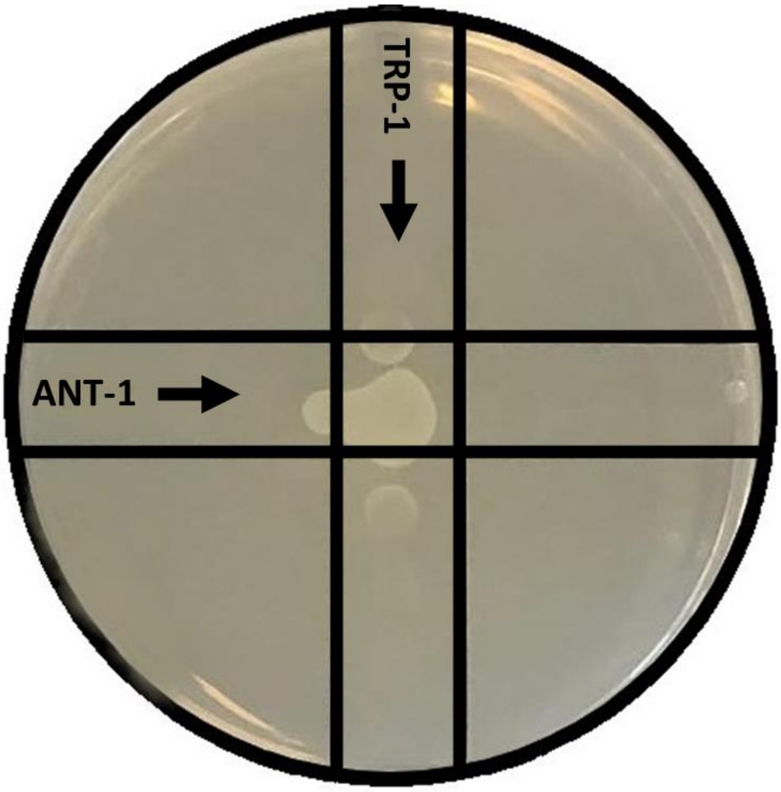

Figure S1: Growth behavior of ANT-1 (left to right), TRP-1 (top to bottom) and co-culture (middle square and in close proximity around) on MM + 0.5 % glucose for 24 h at 37 °C. Cross-feeding and therefore growth is shown where both auxotrophic strains (ANT-1 and TRP-1) meet in the middle. Outside the cooperating region no growth could be detected, proving the strains' auxotrophy.

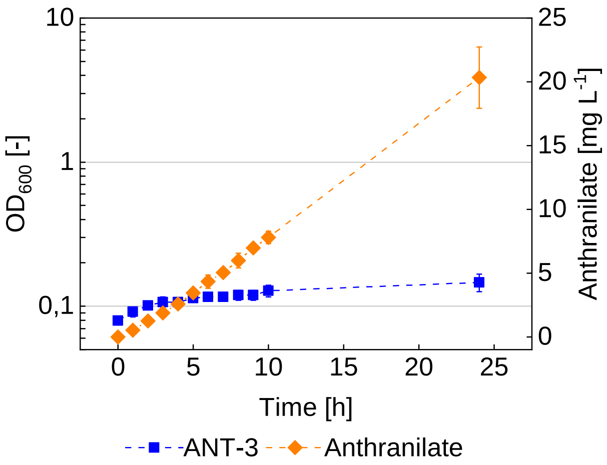

Figure S2: Course of OD$_{600}$ and extracellular anthranilate concentration during cultivation of ANT-3 in shaking flasks without addition of the auxotrophic metabolite tryptophan. The cultivation conditions were as specified in the material and method section 2.2.1. The error bars indicate the standard deviation of the biological replicates.

Table S1: List of primer used in this study

Primer	Sequence 5‘-3‘	PCR template	PCR product
trpD-F	GTATCAGGCGCAGACGCTTAG	*E. coli* LJ110 wildtype	5’trpD‘-linker
trpD-L1	AGGAGACTTTCTGATGGCGGTGCCGCCAGTACCGACGATATC		
trpD-L2	CACCGCCATCAGAAAGTCTCCTGTTTATAGTCCGGAACTGGTGCTG	*E. coli* LJ110 wildtype	3’trpD‘-linker
trpD-R	CACGGTTTTCTTCCGGTGTTCC		
trpD-F	GTATCAGGCGCAGACGCTTAG	5‘-trpD‘-linker + linker-trpD‘-3‘	5‘-trpD‘-linker-trpD‘-3‘ → ΔtrpD
trpD-R	CACGGTTTTCTTCCGGTGTTCC		
trpE-F	GCCTGCTGCTGGTAGACAG	*E. coli* LJ110 wildtype	5’trpE‘-linker
trpE-L1	AGGAGACTTTCTGATGGCGGTGCCATCAGCGTTTCAGCGAGAT		
trpE-L2	CACCGCCATCAGAAAGTCTCCTCTCGACAGCCGTATTGAACTGG	*E. coli* LJ110 wildtype	3’trpE‘-linker
trpE-R	CGAGCGGATCACAATGCAGG		
trpE-F	GCCTGCTGCTGGTAGACAG	5‘-trpE‘-linker + linker-trpE‘-3‘	5‘-trpE‘-linker-trpE‘-3‘ → ΔtrpE
trpE-R	CGAGCGGATCACAATGCAGG		
tnaA-F	ATTATGTAATGGAAAACTTTAAACATCTCCCTGAACCGTTCCG CATTCGTGTTGTTGTGTAGGCTGGAGCTGCTTCG	pCO1	tnaA‘-FRT-Km^{R}-FRT-tnaA‘ → ΔtnaA
tnaA-R	TTTTGCGGTGAAGTGACGCAATACTTTCGGTTCGTACGTAAAGGTTAATCCTTTAACATATGAATATCCTCCTTAGTTC		
trpR-F	ATGGCCCAACAATCACCCTATTCAGCAGCGATGGCAGAACAGCGTCACCAGGAGTTGTGTAGGCTGGAGCTGCTTCG	pCO1	trpR‘-FRT-Km^{R} -FRT-tnaA‘ → ΔtrpR
trpR-R	TCAATCGCTTTTCAGCAACACCTCTTCCAGCCACTGGCGCAGCTCGACGGGCGCGGCCATATGAATATCCTCCTTAGTTC		

Stoichiometric model of *E. coli* used for flux balance analyses (FBA)

The model stoichiometry was based on the *E. coli* reference model used by [1], which is essentially based on previously constructed models [2,3]. The *E. coli* biomass composition was derived from [4].

In contrast to the *E. coli* model used by [1], the merged reactions of chorismate synthesis and tryptophan synthesis were replaced by a detailed shikimate pathway and chorismate-based tryptophan synthesis pathway, including corresponding metabolites [5]. Transport reactions were implemented for relevant metabolites of this refinement. Furthermore, a hypothetical production reaction including transport was introduced, which branches equimolar from tryptophan. This is intended to represent hypothetical tryptophan-derived production scenarios. Furthermore, transport fluxes were selected as reversible (except for acetate, biomass and the implemented tryptophan-derived product). Unused amino acid transport steps were not included. Additionally, unbalanced external metabolites were removed. The model reconstruction and the flux balance analysis (FBA) were performed using the COBRA toolbox [6,7].

The model structure is given in table S2. The co-culture flux distributions were examined via sequential FBA simulations, enabling the specification of necessary interacting fluxes as constraints. The key constraints and objective functions used for the FBA assessments are given in table S3. An outline of important FBA results is given in table S4

Table S2: Stoichiometric *E. coli* model used in this work.

Name	Reaction equation
**Reactions**	
accADBC_fabD	ATP + Acetyl-CoA + ACP + HCO3 <=> ADP + Orthophosphate + h + CoA + Malonyl-ACP
AceA	Isocitrate <=> Succinate + Glyoxylate
AceB	H2O + Acetyl-CoA + Glyoxylate <=> h + CoA + L-Malate
ACK	ADP + Acetyl_phosphate <=> ATP + Acetate
ADK	ATP + AMP <=> 2 ADP
AHyd	ATP + H2O -> ADP + Orthophosphate + h
AKDH	2-Oxoglutarate + NAD + CoA -> Succinyl-CoA + NADH + CO2
AMPS	ATP + L-Aspartate + IMP -> ADP + Orthophosphate + h + AMP + Fumarate
AicarS	5 ATP + 4 H2O + L-Aspartate + 2 L-Glutamine + Glycine + CO2 + PRPP + 10-Formyltetrahydrofolate -> 5 ADP + 7 Orthophosphate + 9 h + 2 L-Glutamate + Fumarate + AICAR + Tetrahydrofolate
AkiS	2 h + 2 Pyruvate + NADPH -> H2O + CO2 + 3-Methyl-2-oxobutanoate + NADP
AlaR	L-Alanine <=> D-Alanine
AlaS	L-Glutamate + Pyruvate <=> L-Alanine + 2-Oxoglutarate
Aldo	D-Fructose_1,6-bisphosphate <=> Glycerone_phosphate + D-Glyceraldehyde_3-phosphate
ANTPRT	PRPP + ant -> Pyrophosphate + n5pdrant
ANTS	L-Glutamine + Chorismate -> h + L-Glutamate + Pyruvate + ant
ArgS	ATP + H2O + L-Aspartate + L-Ornithine + Carbamoyl_phosphate <=> 3 Orthophosphate + 4 h + L-Arginine + AMP + Fumarate
AsnS	ATP + H2O + L-Aspartate + NH4 -> 2 Orthophosphate + h + L-Asparagine + AMP
AspS	L-Glutamate + Oxaloacetate <=> L-Aspartate + 2-Oxoglutarate
CarpS	2 ATP + 2 H2O + L-Glutamine + CO2 -> 2 ADP + Orthophosphate + 3 h + L-Glutamate + Carbamoyl_phosphate
CDPK	ADP + CTP <=> ATP + CDP
CHORS	5o3pshi -> Orthophosphate + Chorismate
CMPK	ADP + CDP <=> ATP + CMP
CSA	H2O + Oxaloacetate + Acetyl-CoA <=> h + CoA + Isocitrate
CTPS	ATP + H2O + L-Glutamine + UTP -> ADP + Orthophosphate + 2 h + L-Glutamate + CTP
cynT	H2O + CO2 <=> h + HCO3
CysS	L-Serine + Acetyl-CoA + H2S -> h + L-Cysteine + Acetate + CoA
DAHPS	H2O + Phosphoenolpyruvate + D-Erythrose_4-phosphate -> Orthophosphate + 2-Dehydro-3-deoxy-D-arabino-heptonate_7-phosphate
dAS	h + ATP + NADPH <=> dATP + H2O + NADP
dCS	h + ATP + NADPH + CDP <=> ADP + dCTP + H2O + NADP
dGS	h + 2 ATP + GMP + NADPH <=> 2 ADP + dGTP + H2O + NADP
DipS	h + ATP + L-Aspartate + L-Glutamate + Succinyl-CoA + Pyruvate + 2 NADPH -> ADP + Orthophosphate + 2-Oxoglutarate + CoA + 2 NADP + LL-2,6-Diaminoheptanedioate + Succinate
dTS	3 ATP + H2O + 2 NADPH + 5,10-Methylenetetrahydrofolate + UDP -> 3 ADP + 2 Orthophosphate + dTTP + Tetrahydrofolate + 2 NADP
ENO	D-Glycerate_2-phosphate <=> H2O + Phosphoenolpyruvate
fabABFGIZ	10 h + 7 NADPH + 3 Malonyl-ACP + Acetoacetyl-ACP <=> 4 H2O + 3 CO2 + 7 NADP + 3 ACP + 2-trans-Decenoyl-ACP
fabABGIZ_161	9 h + 6 NADPH + 3 Malonyl-ACP + 2-trans-Decenoyl-ACP <=> 3 H2O + 3 CO2 + 6 NADP + 3 ACP + cis-Hexadecenoyl-ACP
fabABGI_140	3 h + 2 NADPH + Malonyl-ACP + Dodecanoyl-ACP <=> H2O + CO2 + 2 NADP + ACP + Tetradecanoyl-ACP
fabAFGI_120	3 h + NADH + NADPH + Malonyl-ACP + Decanoyl-ACP <=> H2O + CO2 + NAD + NADP + ACP + Dodecanoyl-ACP
fabAFGI_160	3 h + 2 NADPH + Malonyl-ACP + Tetradecanoyl-ACP <=> H2O + CO2 + 2 NADP + ACP + Hexadecanoyl-ACP
fabAGHI_50	3 h + 2 NADPH + Malonyl-ACP + Propionyl-CoA <=> H2O + CO2 + CoA + 2 NADP + Pentanoyl-ACP
fabBGIZ_150	15 h + 10 NADPH + 5 Malonyl-ACP + Pentanoyl-ACP -> 5 H2O + 5 CO2 + 10 NADP + 5 ACP + Pentadecanoyl-ACP

fabBGIZ_170	3 h + 2 NADPH + Malonyl-ACP + Pentadecanoyl-ACP <=> H2O + CO2 + 2 NADP + ACP + Heptadecanoyl-ACP
fabBGIZ_181	3 h + 2 NADPH + Malonyl-ACP + cis-Hexadecenoyl-ACP <=> H2O + CO2 + 2 NADP + ACP + cis-Octadecenoyl-ACP
fabI_100	h + NADH + 2-trans-Decenoyl-ACP -> NAD + Decanoyl-ACP
fab_BH	h + Acetyl-CoA + Malonyl-ACP <=> CO2 + CoA + Acetoacetyl-ACP
FADHO	FADH2 + CoQ -> CoQH2 + FAD
FATP	0.25 ADP + 0.25 Orthophosphate + 0.25 h + H_out -> 0.25 ATP + 0.25 H2O + H_in
fatty	0.15 cis-Hexadecenoyl-ACP + 0.11 Tetradecanoyl-ACP + 0.04 Dodecanoyl-ACP + 0.35 Hexadecanoyl-ACP + 0.11 Pentadecanoyl-ACP + 0.12 Heptadecanoyl-ACP + 0.12 cis-Octadecenoyl-ACP <=> fatty-ACP
FDH	NAD + Formate <=> NADH + CO2
fThfS	ATP + NADH + CO2 + Tetrahydrofolate -> ADP + Orthophosphate + NAD + 10-Formyltetrahydrofolate
FU	H2O + Fumarate <=> L-Malate
G1PAT	ATP + alpha-D-Glucose_1-phosphate -> ADPglucose + Pyrophosphate
G3PDH	h + NADH + Glycerone_phosphate <=> NAD + sn-Glycerol_3-phosphate
G6PDH	H2O + NADP + beta-D-Glucose_6-phosphate -> 2 h + NADPH + 6-Phospho-D-gluconate
GAPDH	Orthophosphate + NAD + D-Glyceraldehyde_3-phosphate <=> h + NADH + 3-Phospho-D-glyceroyl_phosphate
GMPS	ATP + 3 H2O + L-Glutamine + NAD + IMP -> 2 Orthophosphate + 4 h + L-Glutamate + AMP + GMP + NADH
GluR	L-Glutamate <=> D-Glutamate
GlumS	ATP + L-Glutamate + NH4 <=> ADP + Orthophosphate + L-Glutamine
GlutS	h + 2-Oxoglutarate + NADPH + NH4 -> H2O + L-Glutamate + NADP
GlyS	L-Serine + Tetrahydrofolate <=> H2O + Glycine + 5,10-Methylenetetrahydrofolate
HisS	ATP + 5 H2O + L-Glutamine + 2 NAD + PRPP -> 5 Orthophosphate + 7 h + L-Histidine + 2 NADH + 2-Oxoglutarate + AICAR
HomS	2 h + ATP + L-Aspartate + 2 NADPH <=> ADP + Orthophosphate + 2 NADP + L-Homoserine
ICD	NAD + Isocitrate <=> NADH + CO2 + 2-Oxoglutarate
I3GPS	h + 12cpa1ddr5p -> H2O + CO2 + inglyp
IMPS	AICAR + 10-Formyltetrahydrofolate -> 2 h + H2O + IMP + Tetrahydrofolate
Ileus	2 h + L-Glutamate + L-Threonine + Pyruvate + NADPH -> H2O + L-Isoleucine + CO2 + 2-Oxoglutarate + NADP + NH4
LeuS	H2O + L-Glutamate + NAD + 3-Methyl-2-oxobutanoate + Acetyl-CoA -> h + L-Leucine + NADH + CO2 + 2-Oxoglutarate + CoA
LysS	h + LL-2,6-Diaminoheptanedioate -> L-Lysine + CO2
MDH	NAD + L-Malate <=> h + NADH + Oxaloacetate
meThfS	NADH + CO2 + NH4 + 5,10-Methylenetetrahydrofolate <=> Glycine + NAD + Tetrahydrofolate
MetS	H2O + L-Cysteine + Succinyl-CoA + L-Homoserine + 5-Methyltetrahydrofolate -> h + L-Methionine + CoA + Tetrahydrofolate + Pyruvate + NH4 + Succinate
MurS	7 ATP + 5 H2O + L-Alanine + 2 L-Glutamine + NADPH + D-Alanine + 2 Acetyl-CoA + Phosphoenolpyruvate + LL-2,6-Diaminoheptanedioate + D-Glutamate + 2 beta-D-Fructose_6-phosphate -> 6 ADP + 11 Orthophosphate + 11 h + Murein_subunit + 2 L-Glutamate + AMP + 2 CoA + NADP
myThfS	3 h + 3 NADH + CO2 + Tetrahydrofolate -> 2 H2O + 3 NAD + 5-Methyltetrahydrofolate
NADHO	h + NADH + CoQ + 4 H_in -> NAD + CoQH2 + 4 H_out
OrnS	ATP + H2O + 2 L-Glutamate + NADPH + Acetyl-CoA -> ADP + Orthophosphate + Acetate + 2-Oxoglutarate + CoA + NADP + L-Ornithine
PDH	NAD + CoA + Pyruvate -> NADH + CO2 + Acetyl-CoA
PFK	ATP + beta-D-Fructose_6-phosphate -> ADP + h + D-Fructose_1,6-bisphosphate
PGDH	NADP + 6-Phospho-D-gluconate -> CO2 + NADPH + D-Ribulose_5-phosphate
PGI	beta-D-Glucose_6-phosphate <=> beta-D-Fructose_6-phosphate
PGK	ADP + 3-Phospho-D-glyceroyl_phosphate <=> ATP + D-Glycerate_3-phosphate
PGM	beta-D-Glucose_6-phosphate <=> alpha-D-Glucose_1-phosphate
PGluMu	D-Glycerate_3-phosphate <=> D-Glycerate_2-phosphate

pgsA_pgpA	H2O + sn-Glycerol_3-phosphate + CDP-diacylglycerol <=> Orthophosphate + h + Phosphatidylglycerol + CMP
PheS	h + L-Glutamate + Chorismate -> H2O + L-Phenylalanine + CO2 + 2-Oxoglutarate
PK	ADP + h + Phosphoenolpyruvate -> ATP + Pyruvate
plsBdsA	0.5 CTP + 0.5 sn-Glycerol_3-phosphate + fatty-ACP <=> 0.5 Pyrophosphate + ACP + 0.5 CDP-diacylglycerol
PPase	H2O + Pyrophosphate -> 2 Orthophosphate
PpsA	ATP + H2O + Pyruvate -> Orthophosphate + 2 h + AMP + Phosphoenolpyruvate
PRANTI	n5pdrant -> 12cpa1ddr5p
pssA_psd	L-Serine + CDP-diacylglycerol <=> Phosphatidylethanolamine + CMP + CO2
ProS	h + ATP + L-Glutamate + 2 NADPH -> ADP + Orthophosphate + H2O + L-Proline + 2 NADP
PTA	Orthophosphate + Acetyl-CoA <=> Acetyl_phosphate + CoA
R5PI	D-Ribose_5-phosphate <=> D-Ribulose_5-phosphate
RPE	D-Ribulose_5-phosphate <=> D-Xylulose_5-phosphate
RPPK	ATP + D-Ribose_5-phosphate -> h + AMP + PRPP
sdaA_1	L-Threonine -> NH4 + 2-Oxobutyrate
SDH	Succinate + FAD <=> Fumarate + FADH2
SerS	H2O + L-Glutamate + NAD + D-Glycerate_3-phosphate <=> Orthophosphate + h + L-Serine + NADH + 2-Oxoglutarate
SHID	h + NADPH + 3dshi -> NADP + shi
SHIK	ATP + shi -> ADP + h + shi3p
SUS	ADP + Orthophosphate + Succinyl-CoA <=> ATP + CoA + Succinate
SulRed	4 h + 2 ATP + 4 NADPH + Sulfate -> 2 ADP + 2 Orthophosphate + 2 H2O + 4 NADP + H2S
TA	D-Glyceraldehyde_3-phosphate + Sedoheptulose_7-phosphate <=> D-Erythrose_4-phosphate + beta-D-Fructose_6-phosphate
tdcE_2	CoA + 2-Oxobutyrate <=> Formate + Propionyl-CoA
ThrS	ATP + H2O + L-Homoserine <=> ADP + Orthophosphate + h + L-Threonine
TIS	Glycerone_phosphate <=> D-Glyceraldehyde_3-phosphate
TKa	D-Ribose_5-phosphate + D-Xylulose_5-phosphate <=> D-Glyceraldehyde_3-phosphate + Sedoheptulose_7-phosphate
TKb	D-Glyceraldehyde_3-phosphate + beta-D-Fructose_6-phosphate <=> D-Erythrose_4-phosphate + D-Xylulose_5-phosphate
TRPS	L-Serine + inglyp -> H2O + L-Tryptophan + D-Glyceraldehyde_3-phosphate
Trp_derived_synthesis_flux	L-Tryptophan -> Trp_derived_product
TyrS	L-Glutamate + NAD + Chorismate -> L-Tyrosine + NADH + CO2 + 2-Oxoglutarate
UDPK	ATP + UDP <=> ADP + UTP
UMPK	ATP + UMP <=> ADP + UDP
UMPS	L-Aspartate + PRPP + Carbamoyl_phosphate + 0.5 O2 -> 3 Orthophosphate + h + H2O + UMP + CO2
UO	CoQH2 + 4 H_in + 0.5 O2 -> H2O + CoQ + 4 H_out
ValS	L-Glutamate + 3-Methyl-2-oxobutanoate <=> L-Valine + 2-Oxoglutarate
3DQS	2-Dehydro-3-deoxy-D-arabino-heptonate_7-phosphate -> Orthophosphate + 3dq
3DQD	3dq -> H2O + 3dshi
3PSHI1CVT	Phosphoenolpyruvate + shi3p -> Orthophosphate + 5o3pshi
**Transport**	
PTS	Phosphoenolpyruvate + beta-D-Glucose_e -> Pyruvate + beta-D-Glucose_6-phosphate
T.acetate	Acetate -> Acetate_e
T.ant	ant <=> ant_e
T.Bio	bio -> bio_e
T.cho	Chorismate <=> Chorismate_e
T.co2	CO2 <=> CO2_e
T.h	h <=> h_e
T.H2O	H2O <=> H2O_e
T.nh4	NH4 <=> NH4_e
T.o2	O2 <=> O2_e
T.p	ADP + 2 Orthophosphate + h <=> ATP + H2O + Orthophosphate_e
T.shi	shi <=> shi_e
T.so4	ADP + Orthophosphate + h + Sulfate <=> ATP + H2O + Sulfate_e
T.trp	L-Tryptophan <=> L-Tryptophan_e

T.trp_derived_product	Trp_derived_product -> Trp_derived_product_e
T.3dshi	3dshi <=> 3dshi_e
Ex.acetate	Acetate_e <=>
Ex.ant	ant_e <=>
Ex.Bio	bio_e ->
Ex.co2	CO2_e <=>
Ex.cho	Chorismate_e <=>
Ex.glc	beta-D-Glucose_e <=>
Ex.h	h_e <=>
Ex.nh4	NH4_e <=>
Ex.H2O	H2O_e <=>
Ex.o2	O2_e <=>
Ex.p	Orthophosphate_e <=>
Ex.shi	shi_e <=>
Ex.so4	Sulfate_e <=>
Ex.trp	L-Tryptophan_e <=>
Ex.trp_derived_product	Trp_derived_product_e ->
Ex.3dshi	3dshi_e <=>
**Polymerization reactions**	
BioS_Taymaz_Nikerel	23.1 Protein + 0.7 Mur + 10 H2O + 5.4 DNA + 49.9 RNA + 2.2 Lipid + 8.7 polys <=> bio
DNAPol	2.71 dATP + 2.79 dGTP + 2.71 dTTP + 2.79 dCTP + 10 ATP + 10 H2O <=> 10 ADP + 10 Orthophosphate + 10 h + DNA
LipidPol	31.99 Phosphatidylglycerol + 97.01 Phosphatidylethanolamine <=> Lipid
MurPol	11 Murein_subunit <=> Mur + 20 H2O
ProtPol	1276 ATP + 1276 H2O + 35.84 L-Alanine + 16 L-Arginine + 16 L-Aspartate + 16 L-Asparagine + 5.44 L-Cysteine + 17.92 L-Glutamate + 17.92 L-Glutamine + 27.52 Glycine + 5.44 L-Histidine + 14.72 L-Isoleucine + 29.12 L-Leucine + 17.92 L-Lysine + 7.68 L-Methionine + 10.88 L-Phenylalanine + 13.44 L-Proline + 15.68 L-Serine + 16.96 L-Threonine + 3.52 L-Tryptophan + 8.96 L-Tyrosine + 23.04 L-Valine <=> Protein + 1276 ADP + 1276 Orthophosphate + 1276 h
RNAPol	30 ATP + 30 H2O + 2.88 AMP + 3.54 GMP + 2.38 UMP + 2.2 CMP <=> 30 ADP + 30 Orthophosphate + 30 h + RNA
SPol	10 ADPglucose + alpha-D-Glucose_1-phosphate <=> 10 ADP + 10 h + polys

Table S3: Overview of key settings for the performed flux balance analysis.

A) Co-culture design 1

**Strain 1.I**			**Strain 1.II**		
**Objective Function (max)**	**Constrained fluxes**	**Value**	**Objective Function (max)**	**Constrained fluxes**	**Value**
Chorismate export	Glucose uptake	≤ 10000 µmol (g h)$^{-1}$	Tryptophan derived product export	Glucose uptake	≤ 1000 µmol (g h)$^{-1}$
	Growth	≥ 0.5 h^{-1}		Growth	≥ 0.05 h^{-1}
	Anthranilate synthase (ANTS)	Deletion (= 0)		D 3-Deoxy-D-arabinoheptulosonate 7-phosphate synthase (DAHPS)	Deletion (= 0)
	Tryptophan uptake	≤ Tryptophan demand		Tryptophan export	≥ Tryptophan demand strain 1.I
	ATP-Hydrolysis (Assumed maintenance)	≥ 10 % ATP-synthase flux		ATP-Hydrolysis (Assumed maintenance)	≥ 10 % ATP-synthase flux
				Chorismate uptake	≤ Chorismate export strain 1.I

B) Co-culture design 2

**Strain 2.I**			**Strain 2.II**		
**Objective Function (max)**	**Constrained fluxes**	**Value**	**Objective Function (max)**	**Constrained fluxes**	**Value**
Anthranilate export	Glucose uptake	≤ 10000 µmol (g h)$^{-1}$	Tryptophan derived product export	Glucose uptake	≤ 1000 µmol (g h)$^{-1}$
	Growth	≥ 0.5 h^{-1}		Growth	≥ 0.05 h^{-1}
	Anthranilate phosphoribosyl transferase (ANTPRT)	Deletion (= 0)		Anthranilate synthase (ANTS)	Deletion (= 0)
	Tryptophan uptake	≤ Tryptophan demand		Tryptophan export	≥ Tryptophan demand strain 2.I
	ATP-Hydrolysis (Assumed maintenance)	≥ 10 % ATP-synthase flux		ATP-Hydrolysis (Assumed maintenance)	≥ 10 % ATP-synthase flux
				Anthranilate uptake	≤ Anthranilate export strain 2.I

C) Reference mono-culture

Monoculture			
	**Objective Function (max)**	**Constrained fluxes**	**Value**
	Tryptophan derived product export	Glucose uptake	≤ 1000 µmol (g h)$^{-1}$
		Growth	≥ 0.05 h^{-1}
		ATP-Hydrolysis (Assumed maintenance)	≥ 10 % ATP-synthase flux

Table S4: Overview of essential FBA results. Model stoichiometry can be found in table S2, the constraints used for FBA can be found in table S3. Bold, black: Reactions and transport steps directly associated with the tryptophan synthesis pathway, synthesis steps of relevant building blocks for tryptophan synthesis and metabolic fluxes for the estimation of ATP and NADPH supply. Bold printed, green: Optimized target flux. Fields with a green or red background indicate higher or lower fluxes than in the mono-culture reference. (A) Results Strain 1.I and 2.I, (B) Results mono-culture reference and co-culture strains 1.II and 2.II

A) Fast growing, CHOR/ANT exporting, TRP auxotrophic strains

Name	Strain 1.I	Strain 2.I
	Reaction fluxes [μmol (g h^{-1})]	
**Ex.ant**	0	**2585**
**Ex.cho**	**1847**	0

B) Slow growing, CHOR/ANT auxotrophic, TRP exporting, TRP-derived production strains

Name	Mono-Reference	Strain 1.II	Strain 2.II
	Reaction fluxes [μmol (g h^{-1})]		
AKDH	0	310	0
Aldo	560	0	466
**ANTPRT**	**149**	**925**	**353**
**ANTS**	**149**	**1280**	**0**
**CHORS**	**172**	**0**	**23**
CSA	275	585	275
**DAHPS**	172	**0**	23
ENO	1110	-54	961
**FATP**	**22614**	**36889**	**24676**
FU	249	559	249
**G6PDH**	**758**	**1060**	**785**
GAPDH	1328	940	1383
**GlumS**	**259**	**1390**	**110**
GlutS	655	1431	859
ICD	76	386	76
MDH	448	758	448
PDH	723	1033	723
PFK	560	0	466
**PGDH**	**758**	**1060**	**785**
PGI	237	-65	210
**PGK**	**1328**	**940**	**1383**
PGluMu	1110	-54	961
PheS	13	13	13
PpsA	234	1054	85
R5PI	-434	-994	-529

RPE	324	65	256
**RPPK**	**186**	**962**	**389**
SDH	199	509	199
**SHID**	**172**	**0**	**23**
**SHIK**	**172**	**0**	**23**
**SUS**	**-30**	**280**	**-30**
**SerS**	**218**	**994**	**422**
TA	248	33	140
TIS	543	-18	448
TKa	248	33	140
TKb	-76	-33	-117
TyrS	10	10	10
**TRPS**	**149**	**925**	**353**
**3PSHI1CVT**	**172**	**0**	**23**
**Transport fluxes [μmol (g h^{-1})]**			
**Ex.ant**	**0**	**355**	**-353**
**Ex.cho**	**0**	**-1303**	**0**
**Ex.trp_derived_product**	**145**	**880**	**308**

Reference *E. coli* LJ110 wildtype cultivation and estimation of an OD_{600} - CDW correlation factor

Reference batch cultivations of the E. coli *LJ110* wild-type strain were performed. Procedure and conditions were mainly similar to the specifications given in the material and methods section for single reactor cultivations (section 2.5). In deviation from this, an initial glucose concentration of 25 g L^{-1} was provided with a starting volume of 0.8 L. Furthermore, aeration rate-control was activated after the maximum stirrer speed was reached to keep the set oxygen threshold. The OD_{600} and CDW determination was performed in technical triplicates. For each sampling point replicate, 5 mL of cell suspension was transferred to a prepared glass centrifuge tube (dried at 105°C for at least 48h and weighed after cooling in an exsiccator). The suspension was then centrifuged (2500g, 4°C, 10 minutes) and the supernatant discarded. Subsequently, the cell pellet was washed twice with 0.9% (w/v) NH_4HCO_3. The glass tube including the pellet was then dried at 105 °C for at least 48 h, cooled down in an exsiccator and weighed again.

The results of the cultivation as well as the OD_{600} - CDW correlation factor are shown in figure S3.

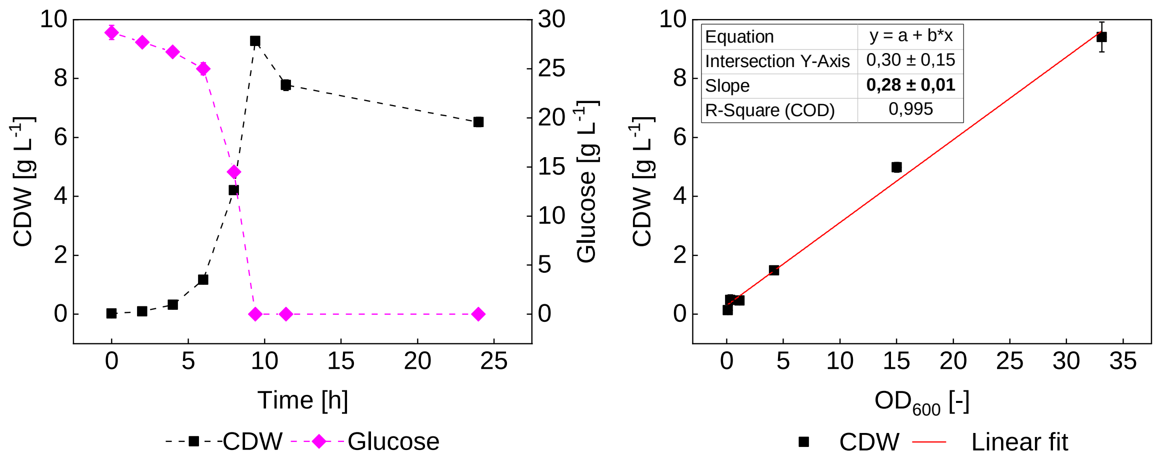

Figure S3: Growth curve and glucose consumption profile of the *E. coli* LJ110 wildtype in a batch cultivation(A) and OD_{600} – CDW correlation factor determination via linear least-square linear regression (slope) (B). The error bars indicate the standard deviation of the biological replicates.

References

[1] Schuhmacher, T., Löffler. M., Hurler, T., Takors, R., Phosphate limited fed-batch processes: impact on carbon usage and energy metabolism in *Escherichia coli*. *J Biotechnol*. 2014; *190*, 96-104.

[2] Chassagnole, C., Noisommit-Rizzi, N., Schmid, J.W., Mauch, K., Reuss, M., Dynamic modeling of the central carbon metabolism of *Escherichia coli*. *Biotechnol Bioeng*. 2002,*79(1)*, 53-73.

[3] Schmid, J. W., Mauch, K., Reuss, M., Gilles, E. D., & Kremling, A., Metabolic design based on a coupled gene expression—metabolic network model of tryptophan production in *Escherichia coli*. *Metab. Eng*. 2004, *6(4)*, 364-377.

[4] Taymaz-Nikerel, H., Borujeni, A. E., Verheijen, P. J., Heijnen, J. J., & van Gulik, W. M., Genome-derived minimal metabolic models for *Escherichia coli* MG1655 with estimated in vivo respiratory ATP stoichiometry. *Biotechnol. Bioeng.* 2010, *107(2)*, 369-381.

[5] Keseler, I. M., Mackie, A., Santos-Zavaleta, A., Billington, R., Bonavides-Martínez, C., Caspi, R., Fulcher, C., Gama-Castro, S., Kothari, A., Krummenacker, M., Latendresse, M., Muñiz-Rascado, L., Ong, Q., Paley, S., Peralta-Gil, M., Subhraveti, P., Velázquez-Ramírez, D. A., Weaver, D., Collado-Vides, J., Paulsen, I., Karp, P. D. The EcoCyc database: reflecting new knowledge about *Escherichia coli* K-12. *Nucleic Acids Res*. 2017; *45(D1)*, 543–550.

[6] Vlassis, N., Pacheco, M.P., Sauter, T., Fast Reconstruction of Compact Context-Specific Metabolic Network Models. *PLoS Comput. Biol*. 2014, 10, e1003424.

[7] Heirendt, L., Arreckx, S., Pfau, T., Mendoza, S.N., et al., Creation and analysis of biochemical constraint-based models using the COBRA Toolbox v.3.0. *Nat. Protoc*. 2019, 14, 639–702.
